# Effect of Dental Chair Backrest Inclination on Mandibular Position Obtained with a Virtual Interocclusal Record at the Maximum Intercuspal Position: A Pilot Clinical Study

**DOI:** 10.3390/dj14090590

**Published:** 2026-09-11

**Authors:** Kawther Ali, Sasiya Bhumpattarachai, Roberto Savignano, Mason Possinger, Joseph Kan

**Affiliations:** 1 Department of Restorative Sciences, College of Dentistry, Kuwait University, Sabah Al Salem University City, P.O. Box 5969, Safat 13060, Shadadiya, Kuwait; 2 School of Dentistry, Loma Linda University, Loma Linda, CA 92350, USA; kellysasiya@gmail.com (S.B.);; 3 Materials Science and Engineering Department, University of Illinois Urbana-Champaign, Urbana, IL 61801, USA

**Keywords:** dental chair inclination, intraoral scanner, mandibular position, maximum intercuspation, virtual interocclusal record

## Abstract

**Background**: This pilot clinical study aimed to evaluate the mandibular position at the maximum intercuspal position (MIP) obtained with a virtual interocclusal record using an intraoral scanner across different dental chair backrest inclinations, in order to establish a protocol for future larger studies. **Methods**: In this single-center pilot study, 10 fully dentate participants underwent maxillary and mandibular full-arch intraoral scans. Virtual interocclusal records at MIP were obtained with an intraoral scanner at three dental chair backrest inclinations (90, 120, and 180 degrees). Conventional vinyl polysiloxane interocclusal records were made at the same inclinations. All scans were printed into physical models and mounted using the conventional records. Digitized casts and intraoral scans were superimposed onto a single common reference, the virtually mounted 90-degree scan, using metrology software. Because all deviations were expressed relative to that reference, the virtual 90-degree condition is zero by construction and was excluded as a data level from inferential models. Separate linear mixed-effects models were fitted for each translation and rotation axis, with participant as a random intercept and recording method, inclination, and their interaction as fixed effects; *p* values were Holm-adjusted across the six axes. Equivalence was explored with two one-sided tests against margins of ±0.5 mm and ±1.0 degrees; because these margins were specified post hoc, the equivalence analysis is exploratory. **Results**: Chair inclination did not detectably affect either recording method: no inclination main effect or method × inclination interaction survived multiplicity correction, and virtual records at 120 and 180 degrees differed by no more than 0.05 mm and 0.08 degrees. Recording method did differ. Conventional records were displaced relative to virtual records by 0.27 mm along the Y axis (95% CI 0.19 to 0.34; Holm-adjusted *p* < 0.001), in the same direction in 9 of 10 participants. The corresponding X-axis offset was 0.23 mm (95% CI 0.06 to 0.40) and did not survive multiplicity correction in the primary analysis (Holm-adjusted *p* = 0.060). The exploratory equivalence criterion was met, that is, the 90% confidence interval fell entirely within the post hoc equivalence margin, for 11 of 18 method × inclination comparisons, rising to 16 of 18 in the sensitivity analysis. **Conclusions**: In this pilot sample, no statistically detectable effect of dental chair backrest inclination between 90 and 180 degrees on the recorded mandibular position at MIP was observed for either recording method in healthy, fully dentate individuals; virtual and conventional records met the exploratory post hoc equivalence criterion in most rotational axes but showed a small, systematic translational offset of approximately 0.23 to 0.31 mm, most clearly along the Y axis. These preliminary findings require confirmation in a larger, prospectively powered and randomized equivalence trial with a prospectively defined equivalence margin.

## 1. Introduction

Capturing the correct locations of occlusal contacts at the maximum intercuspal position (MIP) with an interocclusal record is important for the accurate mounting of a patient’s maxillary and mandibular models [1,2]. The maximum intercuspal position (MIP) is the complete intercuspation of the opposing teeth independent of condylar position, as defined in The Glossary of Prosthodontic Terms [1]. Accurate records lead to precise occlusal analysis, diagnosis, and prostheses [2]. Clinically, MIP is influenced by factors that include body posture, head position, dental chair backrest inclination, the psychological status of the patient, diurnal variations, and masticatory muscle size, strength, force, and the pressure exerted during occlusion and mastication [3,4,5]. For example, Berry and Singh concluded that occlusal contacts change diurnally as a result of the physical state of the masticatory muscles and of the patient [3].

Mandibular posture is not static; it is continuously modulated by the interaction of gravity, masticatory muscle tone, and head and body position. Changes in head and body posture alter the resting length and activity of the elevator and suprahyoid muscles, which can in turn shift the rest position and the trajectory of closure into MIP [3,4]. Variations in the distribution and intensity of occlusal contacts have likewise been linked to muscle physiology and the magnitude of closing force [5]. When a patient is reclined, the combined influence of gravity on the mandible and on the surrounding musculature may therefore translate into measurable changes in the registered jaw relationship.

Conventionally, a polyvinyl siloxane (PVS) material is injected between the teeth and the patient is asked to close into MIP to obtain an interocclusal record [6]. With recent advances in digital technology, digital (virtual) interocclusal records are now possible [6]. These virtual interocclusal records are made by scanning the buccal and labial tooth surfaces while the patient is in MIP [6]; the scanner’s software algorithm then calculates the distance between the opposing arches and registers the maximum intercuspal position and mandible position virtually [7,8].

Various in vitro studies have evaluated the accuracy of virtual interocclusal records and concluded that it is comparable to conventional techniques [6,9,10,11,12]. Iwaki et al. concluded that virtual interocclusal records are accurate when a single tooth is prepared but not when the patient has multiple prepared or missing teeth [13]. Arslan et al. confirmed this finding, as the highest interocclusal record precision was found in fully dentate patients with no prepared teeth [14]. Solaberrieta et al. reported that the validity of virtual records is confirmed and that their accuracy depends on the scanning technique and the section used to obtain the record; they reported that extending the right or left buccal interocclusal scan to the anterior region, or overlapping the right and left buccal interocclusal scans, gives the most accurate results [9,15]. However, other authors have reported opposing results, in which quadrant scans articulated the virtual models more accurately than full-arch scans [16].

Previous studies have investigated the effect of dental chair backrest inclination on the conventional registration of mandibular position [17,18]. Coelho et al. recruited 10 young participants with complete, healthy dentition to register occlusal contacts at 90, 120, and 180 degrees of dental chair backrest inclination and found a difference in mandibular position as a result of the inclination [17]. Tripodakis et al. tested the mandibular position of 11 participants in supine and sitting positions and found that centric relation and occlusion were not affected by body posture [18].

Studies have addressed the influence of head posture on occlusal contacts recorded at MIP with intraoral scanners and digital occlusal analysis [19], the effect of patient position on digitally recorded interocclusal distance in prepared teeth [20], and discrepancies in the maximal intercuspal position recorded by four intraoral scanners at different dental chair positions [21]. A systematic review and meta-analysis of static virtual articulation has additionally established that intraoral scanners do not reproduce the articulated relationship exactly, reporting a pooled trueness of approximately 244 µm (95% CI 145 to 342) for linear deviations and a pooled precision of approximately 55 µm (95% CI 43 to 66) for 3D deviations in articulated models [22]. Any deviation observed in the present study must therefore be interpreted against a baseline in which measurable error is always present.

What remains less well characterized is the specific combination examined in this study: a full-arch virtual interocclusal record captured at standardized backrest inclinations spanning the full clinical range from upright to fully supine, quantified along all six translational and rotational axes, and compared head-to-head with a conventional record made at the same inclinations in the same participants. Whether scanner alignment algorithms preserve the recorded occlusal relationship across that range, and how any such effect compares in magnitude with the difference between digital and conventional workflows, are the questions this study addresses. They are clinically important because the answer determines whether the chair position chosen for patient comfort or operator access needs to be reconsidered when an interocclusal record is required.

The present pilot study was therefore designed to contribute to this question and to establish a reproducible protocol that can inform the design of future, larger, adequately powered investigations. The aim was to evaluate the mandibular position at MIP obtained with a virtual interocclusal record made with an intraoral scanning system across three different dental chair backrest inclinations, and to compare it with the mandibular position at MIP obtained with a conventional interocclusal record across the same inclinations.

Two null hypotheses and one equivalence hypothesis were specified. The null hypotheses were that (1) dental chair backrest inclination would not affect the mandibular MIP captured with a conventional interocclusal record, and (2) dental chair backrest inclination would not affect the mandibular MIP captured with a virtual interocclusal record. The equivalence hypothesis was that virtual and conventional interocclusal records capture the mandibular MIP within a clinically acceptable margin at each of the three inclinations.

## 2. Materials and Methods

### 2.1. Study Design and Ethical Approval

**Figure 1:**
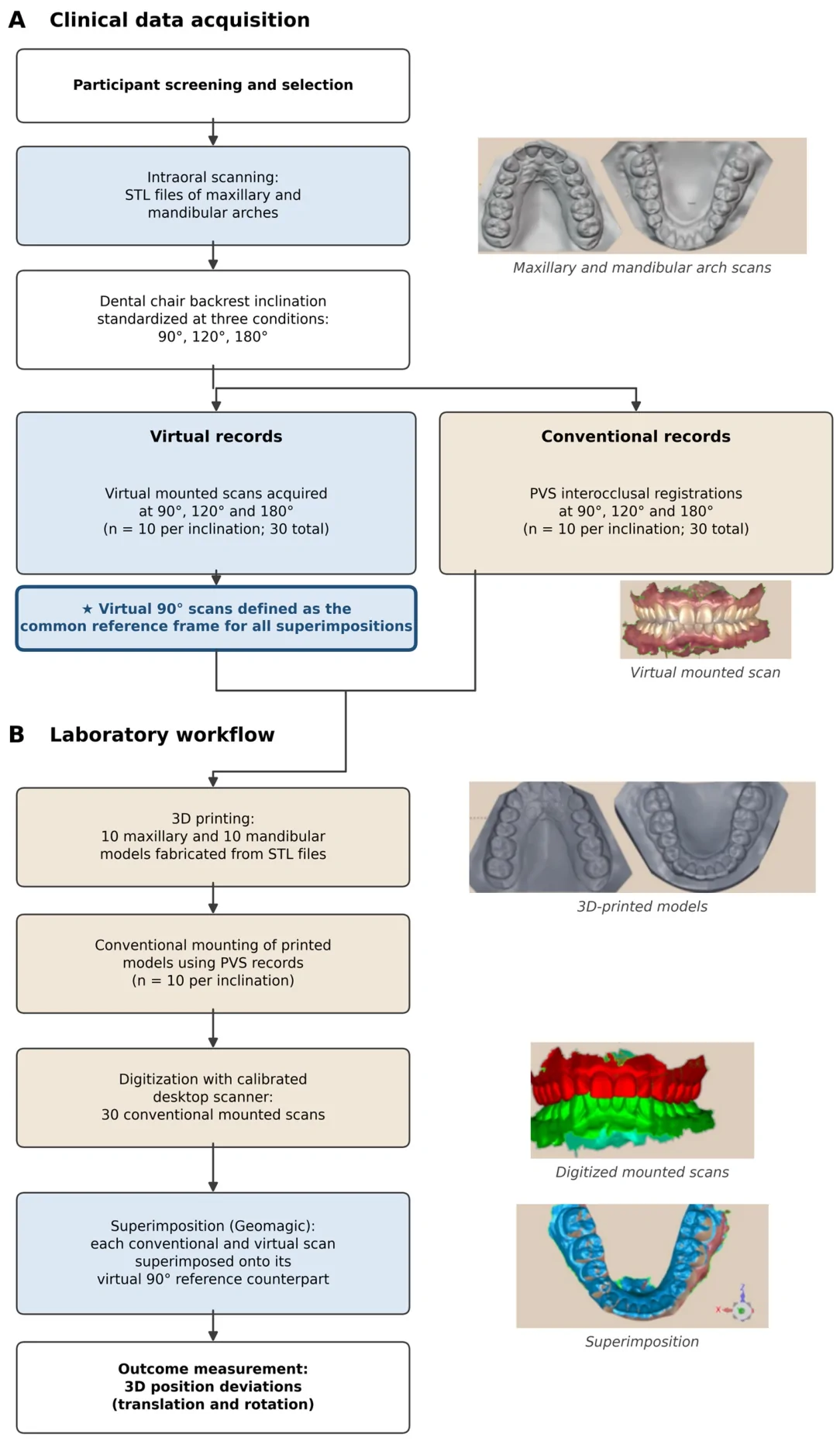
Study design.

This study was a single-center, nonrandomized pilot clinical trial. Approval was obtained from the Loma Linda University Institutional Review Board before the start of the study (IRB No. 5210377). Written informed consent was obtained from all participants. Figure 1 shows the study design.

### 2.2. Sample Size Calculation

An a priori power analysis was performed with G*Power (version 3.1.9.7; Heinrich-Heine-Universität Düsseldorf, Düsseldorf, Germany) before recruitment. The calculation was based on the large effect of dental chair backrest inclination on mandibular position reported by Coelho et al. [17], who detected a clinically meaningful difference of approximately 1.4 mm between the 90- and 180-degree positions. For the within-participant contrasts reported here, 10 participants provide 80% power at α = 0.05 to detect a standardized paired effect of dz = 1.00, which is a large effect; the study was not powered to detect small or moderate ones. Accordingly, this study is reported as a pilot to establish a reproducible measurement protocol and to generate the variance estimates needed to design an adequately powered trial, not to establish that the methods agree.

### 2.3. Participants

Dental students at the school of dentistry were invited by email to volunteer for low-risk dental research involving intraoral scanning. Interested students attended a screening appointment that consisted of providing informed consent and receiving an oral soft- and hard-tissue examination. Ten dental students were selected based on the following inclusion criteria: male and female participants ≥18 years of age; able and willing to sign the informed consent form; in good general and oral health; dentate with at least 24 teeth; Angle Class I molar and canine relationship; and no anterior or posterior crossbite. Students were excluded if they had systemic debilitating diseases, ataxia, trismus, neuromuscular disturbances, temporomandibular joint disorders, limited mouth opening, or advanced periodontal disease with noticeable mobility or fremitus. The total number of participants was 10.

### 2.4. Standardization of Dental Chair Inclination

The interocclusal record of each participant was registered at three dental chair backrest inclinations: 90 degrees (backrest perpendicular to the floor), 120 degrees, and 180 degrees. These inclinations were selected because they represent the range in which a patient is normally seated during a dental visit, depending on the procedure performed. The dental chair inclination was standardized with a protractor positioned at the intersection between the dental chair backrest and its seat. The angle was measured and confirmed by a single trained operator immediately before each interocclusal record was made to ensure that the target inclination of 90, 120, or 180 degrees was reproduced for every record. Head position was standardized visually. Before each record the operator confirmed that the participant’s head rested against the chair headrest in a neutral position, with no visible flexion or extension of the neck relative to the trunk, and participants were instructed to keep the head still. Chair inclinations and recording methods were applied in a fixed order (90, then 120, then 180 degrees; virtual record before conventional record) for every participant.

### 2.5. Intraoral Scanning and Virtual Interocclusal Records

For each participant, maxillary and mandibular scans were made with an intraoral scanner (3Shape TRIOS 3; 3Shape A/S, Copenhagen, Denmark) using a standardized scanning path performed by one experienced operator. Scanning started at the right posterior teeth in a zigzag motion, moving the scanner tip from the buccal surface to the occlusal surface and then to the lingual surface of each tooth. The scanner tip was then advanced anteriorly to the next tooth, capturing the lingual, occlusal, and buccal portions in turn. This process was repeated as the scanner tip moved anteriorly and crossed to the left posterior teeth so that the whole arch was captured. This zigzag path was adopted because of its practicality, acceptable trueness, and precision [23].

Any missing area was rescanned to complete the mesh according to manufacturer recommendations, and a scan was considered complete once all dental surfaces were captured and no major deficiencies were present. Each participant’s maxillary and mandibular scans were saved as standard tessellation language (STL) files.

At the 90-degree inclination, virtual interocclusal records (VR90) were obtained with the intraoral scanner using a standardized scanning protocol. The protocol began midway between the buccal surfaces of the right posterior maxillary and mandibular quadrants, starting at the most distal molar and ending at the canine, with the scanner tip positioned perpendicular to the buccal surfaces of these quadrants until both scans snap into position and the jaws align according to the manufacturer recommendations; the procedure was repeated on the left side. The virtual interocclusal record scans were exported in STL format and served as the control group. The maxillary and mandibular intraoral scans of each participant were duplicated, and virtual interocclusal records were made at 120 degrees (VR120) and 180 degrees (VR180) following the same standardized protocol; the virtually mounted scans were exported in STL format and represented the first (VR120) and second (VR180) experimental groups with 10 scans each (*n* = 10).

MIP closure was standardized by verbal instruction and rehearsal. Before each recording session the procedure was explained and the participant was asked to close fully into maximum intercuspation and to open again several times in succession, so that the closure became familiar and repeatable before any record was captured.

### 2.6. Conventional Interocclusal Records

Two interocclusal records (right and left) were captured for each participant with a vinyl polysiloxane material (Vanilla Bite; Henry Schein Dental, Melville, NY, USA) at the 90-, 120-, and 180-degree inclinations. Before the records were made, participants received verbal guidance and rehearsal to standardize their MIP closure.

At each inclination the procedure was performed three times. The three records were inspected visually for complete and consistent seating on the teeth and for the absence of perforation or distortion, and if at least two of the three records matched during mounting, that record was considered verified, and the corresponding mounted casts were carried forward for desktop scanning.

### 2.7. Model Fabrication and Printing Conditions

All maxillary and mandibular intraoral scans were printed by a single trained operator using a model resin (SprintRay Study Model White 2; SprintRay, Los Angeles, CA, USA) and a calibrated digital light-processing 3D printer (SprintRay Pro 95; SprintRay, Los Angeles, CA, USA). The printer was calibrated according to the manufacturer’s protocol before fabrication. Each model was post-processed (washed and light-cured) following the manufacturer’s instructions. To minimize dimensional change, all printed models were scanned immediately after post-processing. A total of 10 maxillary and 10 mandibular printed models were fabricated.

### 2.8. Mounting and Digitization

An articulator (Panadent 1620 AR; Panadent, Colton, CA, USA) and a mounting table were used to mount the maxillary casts. The mandibular casts were then mounted to the corresponding maxillary casts using the conventional interocclusal records obtained at 90 degrees, and the mounted casts were scanned with a calibrated desktop scanner (3Shape D900L; 3Shape A/S, Copenhagen, Denmark). These STL files represented the third experimental group (CR90). The mandibular casts were subsequently remounted using the conventional records obtained at 120 and 180 degrees and were rescanned with the same calibrated desktop scanner, yielding STL files that represented the fourth (CR120) and fifth (CR180) experimental groups. A total of 10 digitized mounted casts were obtained per position (*n* = 10). All casts were mounted by one experienced operator, using for each inclination the single verified interocclusal record selected as described in Section 2.6.

### 2.9. Superimposition, Measurement, and Reliability

**Figure 2:**
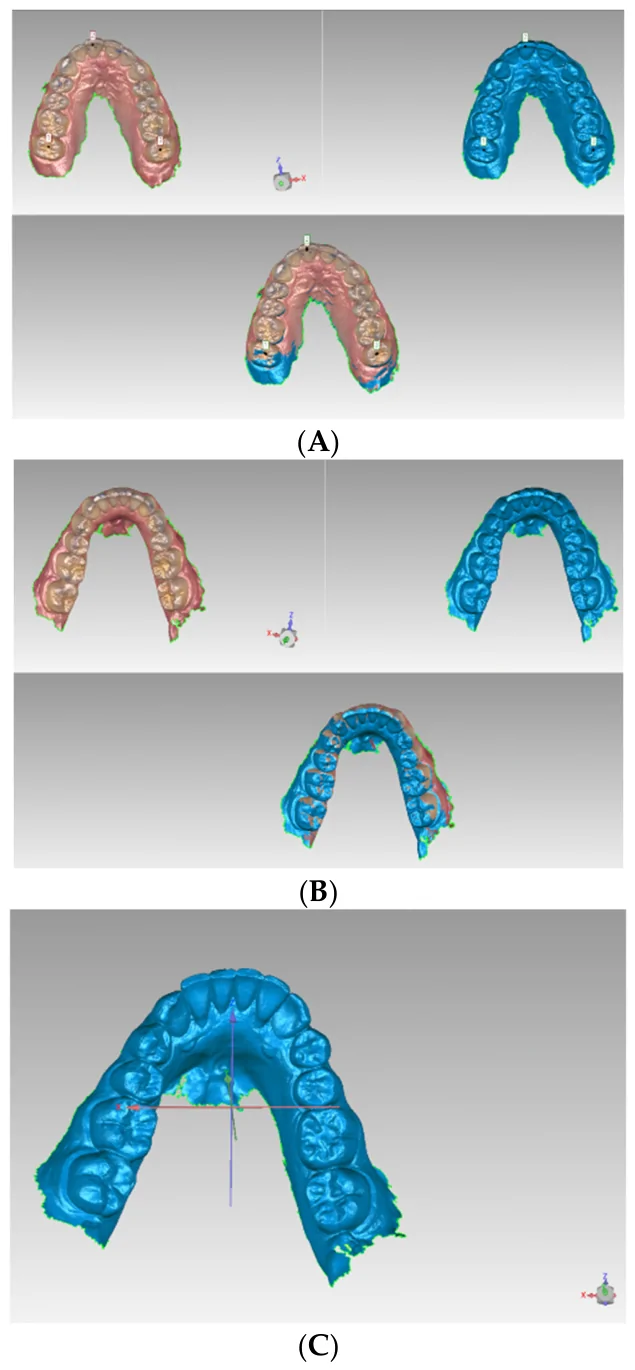
Superimposition and measurement of 3D positional deviations in the metrology software. (**A**) Superimposition of the virtually mounted control scan and the test digital scan by merging points on the maxillary scan. (**B**) The mandibular scan was aligned to the corresponding maxillary scan and then superimposed. (**C**) The 3D positional deviations were analyzed in both translation and rotation along the X, Y, and Z axes.

The STL files of the control and experimental groups were imported into a 3D metrology software program (Geomagic Control X version 2017; 3D Systems, Rock Hill, SC, USA). All scans from the experimental groups were superimposed onto the scans from the control group and aligned using the best-fit algorithm (full surface to full surface). The maxillary scan was used as the reference for best-fit alignment. The mandibular scan was then superimposed based on the aligned maxilla. Deviations in both rotation and translation were analyzed along the X, Y, and Z axes between the reference mandibular scans and the experimental groups (Figure 2). The X axis represented mesiodistal deviation, the Y axis represented occlusal deviation, and the Z axis represented anteroposterior (forward–backward) deviation. All superimpositions, alignments, and deviation measurements were performed by one experienced operator.

To limit random measurement error arising from the superimposition procedure, each participant’s scans were superimposed and measured three times and the mean of the three measurements was carried forward.

### 2.10. Statistical Analysis

All positional deviations were expressed relative to a single common reference: the virtually mounted scan obtained at 90 degrees. The virtual record at 90 degrees is consequently identically zero by construction rather than by observation. A condition with zero variance violates the assumptions of any conventional linear model and is structurally dependent on every other condition, so the virtual 90-degree condition was treated as the reference frame and excluded as a data level from all inferential models. It is reported descriptively only.

The quantities analyzed are therefore differences, not absolute positions, and each has a specific meaning. A virtual deviation at 120 or 180 degrees (VR120, VR180) is the displacement of the mandible recorded by the scanner at that inclination relative to the mandible recorded by the scanner at 90 degrees, and isolates the effect of inclination on the virtual record alone. A conventional deviation (CR90, CR120, CR180) is the displacement of the mandible in the physically mounted casts, articulated with the conventional record made at that inclination, relative to the same virtual 90-degree reference.

Translation is measured in millimeters and rotation in degrees, and the two quantify different clinical phenomena; they were therefore not combined as levels of a single factor. Separate linear mixed-effects models were fitted to each of the six outcomes (translation and rotation along the X, Y, and Z axes). The primary model for each outcome included recording method (conventional, virtual), chair inclination (120, 180 degrees), and their interaction as fixed effects, with participant as a random intercept. This is the portion of the design in which both recording methods are observed and the interaction is estimable. Fixed effects were tested by likelihood-ratio tests against nested models. A second mixed-effects model, restricted to conventional records, tested the effect of inclination across all three levels (90, 120, and 180 degrees), and the effect of inclination on virtual records was tested by the within-participant contrast between the 120- and 180-degree records.

The difference between recording methods at each inclination was additionally estimated as a within-participant paired contrast, reported as a model-estimated mean difference with a 95% confidence interval and a standardized effect size (Cohen’s dz). Because six outcomes were analyzed, *p* values were adjusted by the Holm–Bonferroni procedure within each family of tests; unadjusted and adjusted values are both reported. Model residuals were inspected graphically and tested with the Shapiro–Wilk test, and Wilcoxon signed-rank tests were computed alongside every parametric contrast as a distribution-free check.

Because a nonsignificant difference cannot demonstrate that two methods agree, equivalence between recording methods was assessed by two one-sided tests (TOST). Equivalence was concluded when the 90% confidence interval for the method difference lay entirely within the equivalence margin. The convention of reporting equivalence and difference tests together follows Lakens [24] and Lakens et al. [25]. Margins of ±0.5 mm for translation and ±1.0 degrees for rotation were adopted. These lie within the deviation ranges reported between virtual and conventional records by Zimmermann et al. [12] and within the range judged clinically acceptable by Abdulateef et al. [26], and the translational margin corresponds to the 0.5-mm linear tolerance of the American Board of Orthodontics objective grading system [27]. The same translational margin was applied to all three axes because no axis-specific clinical threshold for whole-arch mandibular displacement has been established; the stricter vertical benchmark that has been proposed for interocclusal records is considered in Section 4. These margins were, however, specified after the data had been collected and inspected, and not prospectively. The two one-sided tests are therefore reported as an exploratory analysis within a pilot study, intended to generate a defensible margin and the variance estimates required to design a future, prospectively powered equivalence trial. Throughout the manuscript, a result is described as meeting the exploratory equivalence criterion, or as having a 90% confidence interval that lies within the post hoc equivalence margin, rather than as establishing equivalence formally. Agreement between the two recording methods was additionally described with the Bland–Altman approach [28], pooling the 120- and 180-degree conditions in which both methods are observed. These 19 differences in the primary analysis, and 18 in the sensitivity analysis, arise from only 10 participants, so a participant may contribute more than one difference and the independence assumption underlying the conventional limits of agreement is not satisfied. Within-participant correlation was not modeled, and the standard formulae were applied unchanged; the Bland–Altman results are consequently reported as descriptive and exploratory, and no inference is drawn from them. All inferential conclusions rest on the linear mixed-effects models, which accommodate the repeated measurements through the participant random intercept. A repeated-measures Bland–Altman analysis, estimating the within-participant and between-participant components of the difference variance separately, is recommended for the confirmatory study.

Outlying observations were identified before modelling by a rule applied uniformly to every method × inclination cell. An observation was flagged when its robust standardized score, computed as 0.6745 × (value−median) ÷ median absolute deviation, exceeded 3.5 in absolute value. A robust score was used in preference to one based on the mean and standard deviation because the latter is inflated by the very observations it is meant to detect. Two records were flagged, and both were extreme on all six axes simultaneously rather than on a single axis; no other record exceeded the threshold on more than two axes, which is why these two are described as multivariate outliers. A technical cause was sought for each. No scanning failure, incomplete seating, mounting error, or superimposition failure was documented for either record at the time it was made. The cause therefore remains unestablished. Every analysis was run twice: a primary analysis including all observations, and a sensitivity analysis excluding the two flagged records. Both are reported. Descriptive statistics (means and standard deviations) of translation (mm) and rotation (degrees) are reported for all conditions. Analyses were performed with R (version 4.3.0; R Foundation for Statistical Computing, Vienna, Austria) and Python (version 3.11, with statsmodels 0.14), with α = 0.05.

## 3. Results

A total of 10 participants were enrolled and all completed the protocol. Mean age was 33.3 years (SD 5.7; range 26–45), and the sample comprised 2 women and 8 men. All participants met the stated inclusion criteria, with complete dentitions of at least 24 teeth, Angle Class I molar and canine relationships, and no anterior or posterior crossbite.

Two records proved to be multivariate outliers. The virtual 180-degree record of participant 8 deviated by 1.39 mm, −4.59 degrees, and −6.14 degrees in the X-translation, Y-rotation, and Z-rotation axes, respectively, whereas every other participant fell within approximately ±0.5 on all six axes; this single record accounts for the entire mean Z-rotational deviation at 180 degrees, which falls from −0.71 degrees (SD 2.05) to −0.04 degrees (SD 0.30) when it is excluded. The conventional 90-degree record of participant 5 showed a Z-axis translation of +5.61 mm against a range of −0.07 to +0.31 mm in all other participants; excluding it changes the mean Z translation of the conventional 90-degree condition from +0.64 mm (SD 1.75) to +0.09 mm (SD 0.12).

In the primary analysis, Shapiro–Wilk tests of model residuals indicated departures from normality on five of the six outcomes (*p* < 0.05), reflecting the two outlier records. In the sensitivity analysis four of the six outcomes satisfied the assumption, and non-normality persisted only for Z translation and X rotation (both *p* < 0.001), traceable to two further individual observations: participant 5’s conventional 120-degree Z translation of −0.75 mm and participant 2’s conventional 180-degree X rotation of 3.05 degrees. Wilcoxon signed-rank tests were computed alongside every parametric contrast and agreed in direction and in significance status throughout; for the principal Y-translation contrasts, the distribution-free *p* values were 0.002, 0.004, and 0.008 at 90, 120, and 180 degrees, respectively. The parametric results are therefore reported as primary.

Chair inclination did not detectably affect the recorded mandibular position by either method. In the per-axis mixed-effects models, no main effect of inclination and no method × inclination interaction survived Holm correction on any axis (all adjusted *p* ≥ 0.98; Table 1). Restricting the model to conventional records and testing all three inclinations gave the same result: the smallest unadjusted *p* value was 0.041 for Y rotation (0.027 in the sensitivity analysis), which does not survive correction for six outcomes (adjusted *p* = 0.245 and 0.160 respectively; Table 2). Three confidence intervals for the 90- versus 180-degree contrast excluded zero before correction—X translation (−0.18 mm, 95% CI −0.32 to −0.03), X rotation (+0.48 degrees, 95% CI +0.02 to +0.95), and Y rotation (+0.16 degrees, 95% CI +0.00 to +0.32).

The virtual record was particularly stable across inclination. The within-participant contrast between the 120- and 180-degree virtual records was nonsignificant on all six axes (all adjusted *p* = 1.00), and in the sensitivity analysis every mean difference fell below 0.05 mm or 0.08 degrees, with all confidence intervals contained within ±0.39 (Table 3).

Recording method, by contrast, did produce a systematic difference, and the Y translation axis carries it most clearly. Conventional records were displaced relative to virtual records by +0.27 mm along the Y axis (95% CI 0.19 to 0.34; unadjusted and Holm-adjusted *p* < 0.001). The offset ran in the same direction in 9 of 10 participants at 120 degrees (mean +0.25 mm, dz = 2.00, Wilcoxon *p* = 0.004) and was significant at all three inclinations. The X axis showed an offset of the same sign and comparable size, +0.23 mm (95% CI 0.06 to 0.40), but at unadjusted *p* = 0.012 it did not survive Holm correction across the six outcomes (adjusted *p* = 0.060) and is reported as suggestive rather than established. Excluding the two outlier records strengthened both effects, and under that analysis each is unambiguous (X: +0.31 mm, 95% CI 0.22 to 0.41; Y: +0.25 mm, 95% CI 0.18 to 0.31; both Holm-adjusted *p* < 0.001). No method effect was detected on the Z translation axis or on any rotational axis after correction in either analysis (Table 1).

Descriptive statistics for the conventional interocclusal records at each inclination are given in Table 2, together with the mixed-effects estimates of the inclination contrasts.

Descriptive statistics for the virtual interocclusal records are given in Table 3. The virtual 90-degree condition is the reference frame and is zero by construction; it is shown for completeness but contributes no variance and was not used in any inferential model.

Descriptive values for both recording methods at each inclination, together with the Holm-adjusted method effect on each axis, are summarized in Table 4. Exploratory equivalence testing was more informative than the significance tests alone (Table 5). Using the post hoc margins of ±0.5 mm and ±1.0 degrees, the 90% confidence interval for the method difference fell entirely within the margin for 11 of the 18 method × inclination comparisons in the primary analysis and for 16 of 18 in the sensitivity analysis. The exploratory criterion was met for 6 of the 9 rotational comparisons in the primary analysis and 8 of 9 in the sensitivity analysis, and for every comparison at 120 degrees in both analyses. Because the margins were defined post hoc, these results show that an exploratory equivalence criterion was met and do not establish equivalence formally.

Agreement between the two recording methods was additionally described by the Bland–Altman method [28], pooling the 120- and 180-degree conditions in which both methods are observed (Table 6). In the primary analysis the bias was +0.25 mm for X translation and +0.27 mm for Y translation, with 95% limits of agreement of −0.53 to +1.03 mm and −0.10 to +0.64 mm respectively. Excluding the two outlier records narrowed the limits: −0.14 to +0.79 mm for X translation, −0.05 to +0.55 mm for Y translation, and −0.70 to +0.55 mm for Z translation. For rotation the corresponding limits were −1.77 to +1.83 degrees about the X axis, −0.59 to +0.78 degrees about the Y axis, and −0.83 to +0.73 degrees about the Z axis, with biases of +0.03, +0.09, and −0.05 degrees respectively. Because the pooled differences come from 10 participants rather than 19 independent observations, these limits of agreement are not adjusted for within-participant correlation and are presented descriptively only.

## 4. Discussion

The authors failed to reject the first null hypothesis. No statistically detectable effect of dental chair backrest inclination on the mandibular position at MIP captured with conventional interocclusal records survived correction for multiple outcomes. This should be stated carefully: the data are compatible with no effect, but with 10 participants they are also compatible with effects up to roughly 0.17 mm and 0.95 degrees, the upper bounds of the confidence intervals for the 90- versus 180-degree contrasts. The finding contrasts with previous reports that the mandibular position at MIP is influenced by body posture, head position, and dental chair backrest inclination [3,4,5,17]. Coelho et al. investigated chair backrest inclinations by recruiting 10 patients and recording their occlusal contacts at 90, 120, and 180 degrees with an auto-polymerizing methyl methacrylate device; they reported a significant difference of 1.4 mm between the 90- and 180-degree positions and recommended making the record at 90 degrees [17]. An effect of that size would have been detected here, so the present data do provide evidence against a difference as large as 1.4 mm, even though they cannot exclude smaller ones. The 1.4-mm difference reported by Coelho et al. [17] was substantially larger than any inclination-related deviation in this study. Several methodological differences may explain the discrepancy. Coelho et al. registered occlusal contacts with a rigid auto-polymerizing methyl methacrylate device [17], whereas the virtual record here relied on the scanner’s alignment algorithm, which may absorb small positional changes; if so, the algorithm’s stability across inclination is a property of the software as much as of the patient, and this should be borne in mind when generalizing to other scanners and other software versions. Moreover, the two recording materials behave differently. Auto-polymerizing methyl methacrylate polymerizes over an extended interval with appreciable exotherm and polymerization shrinkage, during which the mandible may drift; vinyl polysiloxane sets more rapidly and with lower dimensional change. Some portion of a 1.4-mm difference may therefore be attributable to the recording medium and its setting behavior rather than to backrest inclination itself [30].

The authors also failed to reject the second null hypothesis. The mandibular position at MIP captured with the virtual interocclusal record did not differ detectably across inclinations, and in the sensitivity analysis the 120- versus 180-degree differences were smaller than 0.05 mm and 0.08 degrees on every axis. Our margins of ±0.5 mm and ±1.0 degrees were derived from the deviation ranges reported between recording methods by Zimmermann et al. [12] and Abdulateef et al. [26]. The translational deviation reported in this study is below the reported minimum clinical threshold of 0.5 mm according to the ABO objective grading system [27]. There is no universally accepted threshold for angular deviations, although most reported deviations are below two degrees [31]. Intraoral scanner software is typically programmed with algorithms that align and interdigitate the virtual arches automatically, which offers a plausible mechanism for the insensitivity to inclination observed here [11].

The findings of the present study are broadly consistent with those of recently published studies. The influence of head posture on occlusal contacts recorded at MIP with intraoral scanners and digital occlusal analysis has been examined recently [19]. Ntovas et al. reported that the occlusal contacts digitized for 46 patients were not significantly affected when patients were in an upright or supine position [19]. Opposing findings have also been reported. Nikhil et al. captured the interocclusal separation between the maxillary and mandibular arches virtually and compared it between the upright and reclined positions [20]. They found a mean difference of 0.68 mm between the two chair positions, which is larger than the difference found in the present study [20]. The upright position in their study consistently exhibited greater interocclusal distance values across all participants [20]. Moreover, Revilla-León et al. investigated discrepancies in the maximal intercuspal position recorded by four different intraoral scanners at different dental chair positions [21]; they found statistically significant differences between the scanners and between the dental chair inclinations [21]. However, only one patient was scanned, which limits the generalizability of their results [21].

The systematic review and meta-analysis of static virtual articulation by Morsy and El Kateb [22] reported a pooled trueness of approximately 244 µm for linear deviations, with a 95% confidence interval of 145 to 342 µm, and a pooled precision of approximately 55 µm for 3D deviations in articulated models. Measurable error is therefore always present in virtual articulation, and the relevant question is never whether a deviation exists but whether it exceeds what the technique inherently produces. The translational offset of 0.23 to 0.31 mm reported in this study is of the same order as the pooled trueness of virtual articulation itself. The variability at 180 degrees deserves emphasis. In the primary analysis the standard deviation of the virtual Z-axis rotation at 180 degrees was 2.05 degrees. The 180-degree condition therefore showed greater variability in this sample. Because the study was not designed as a reproducibility study and reliability was not formally established, this observation cannot be taken to show that the supine position is inherently less reproducible than the upright one; it is hypothesis-generating and should be investigated in a larger study designed for that purpose. A method that is unbiased on average but occasionally produces a large error is not equivalent to one that is consistently accurate.

The third question was posed as an equivalence question, and the answer is mixed. The mandibular position at MIP recorded with a virtual or conventional record met the exploratory equivalence criterion of ±1.0 degrees at almost every inclination. Translationally, however, the two methods were not interchangeable. The mandibular position at MIP recorded with a conventional record lay approximately 0.27 mm further along the Y axis than that captured with a virtual record. The X axis showed an offset of the same sign and comparable size, and the Y-axis offset ran in the same direction in 9 of 10 participants. These are small displacements, but they are systematic rather than random. Vinyl polysiloxane is comparatively rigid and may offer slight resistance that prevents the mandible from seating completely against the maxilla [30]. Even though the displacements were below the exploratory margin adopted here (0.5 mm), which corresponds to the linear tolerance of the American Board of Orthodontics objective grading system [27], vertical displacement along the Y axis receives particular attention in prosthodontics because it can lead to occlusal discrepancies and chairside adjustment [30]. No universally accepted threshold for a clinically acceptable vertical discrepancy has been established; however, Vergos and Tripodakis reported that clinically acceptable vertical discrepancies in interocclusal records are below approximately 200 µm [30]. The Y-axis offset observed here, approximately 0.27 mm or 270 µm, lies well inside the ±0.5-mm exploratory margin but exceeds that stricter figure, and the two benchmarks require explicit reconciliation. They do not describe the same quantity. The 200-µm value refers to the vertical error introduced at the level of an individual record between opposing casts as it affects the seating of a restoration, whereas the present measurement is a whole-arch rigid-body displacement of the mandibular cast relative to the maxilla, expressed relative to a virtual reference and accumulated across 3D printing, articulator mounting, and desktop digitization as well as the record itself. No axis-specific clinical threshold has been established for the latter quantity, which is why a single ±0.5-mm margin, the most widely used linear tolerance for occlusal outcomes [27], was applied to all three translational axes. That choice should not be read as a claim that 0.5 mm is the appropriate vertical tolerance. If the 200-µm figure is instead taken as the operative vertical benchmark, the Y-axis difference would not be clinically negligible, and the exploratory equivalence result on the Y axis is accordingly the least secure of those reported here. A prospectively defined, axis-specific vertical margin is therefore required before the two workflows can be regarded as interchangeable in the vertical dimension, and the vertical offset is the quantity that a confirmatory trial should be powered to resolve.

The direction of this offset deserves a comment. Because every deviation is expressed relative to the virtually mounted 90-degree scan, the conventional-minus-virtual difference does not isolate the interocclusal record. It bundles in the entire physical pathway that the conventional arm alone traverses: 3D printing of the casts, mounting on the articulator, and desktop digitization of the mounted casts. Each of those steps carries its own dimensional tolerance, and their accumulation is a more parsimonious explanation for a submillimeter systematic offset than a failure of either recording technique [14,32].

These results are broadly consistent with previous comparisons of virtually and conventionally captured contacts [12,26,32,33]. DeLong et al. compared contacts marked with red film on dental casts with virtual scans of the casts and concluded that aligned virtual casts reproduced articulator contacts accurately [32]. Zimmermann et al. recorded a conventional and a virtual interocclusal record for each of 10 patients and concluded that intraoral scanning captured the static MIP relationship with the same accuracy as the conventional method [12]. Abdulateef et al. concluded that virtual records were clinically acceptable and reported that they were more likely to miss a contact than to record an incorrect one [26]. Notably, the offset observed here falls within the 0.6 to 0.9 mm translational range that Zimmermann et al. themselves reported between the two methods [12].

When the magnitude of deviation is considered, the present results sit within the digital–conventional comparison literature while differing from the chair-inclination literature. The mean deviations observed here (translation ≤ 0.64 mm; rotation ≤ 0.71 degrees in magnitude in the primary analysis, and sensitivity analysis ≤ 0.39 mm and ≤0.35 degrees) are within the ranges reported by Zimmermann et al. [12], who reported rotation of approximately 0.2 to 0.7 degrees and translation of approximately 0.6 to 0.9 mm between virtual and conventional records, and are consistent with the clinical acceptability reported [26,27].

### 4.1. Limitations

This study has several limitations. First, chair inclinations and recording methods were applied in a fixed order without randomization or counterbalancing. Order, muscular fatigue, adaptation to the scanning procedure, and the effect of repeated closure into MIP are consequently confounded with both experimental factors. Randomization or counterbalancing of both factors is essential in any subsequent trial. All participants were healthy dental students with complete dentitions, Angle Class I relationships, and no temporomandibular disorder. Their professional familiarity with intraoral scanning may itself have improved cooperation and closure consistency relative to ordinary patients. The findings cannot be extended to the elderly, partially dentate or edentulous patients, patients undergoing prosthodontic or implant treatment, or patients with temporomandibular disorders, all of whom have mechanisms by which mandibular position might be more sensitive to backrest inclination.

Head position was not standardized objectively. Because the physiological rationale for an effect of inclination depends on gravity and on the resting length and activity of the elevator and suprahyoid muscles, which respond to head position as well as to trunk position, uncontrolled variation in cranio-cervical posture is a plausible source of within- and between-participant variability that this design cannot quantify. Future studies should record a craniometric reference at each inclination.

Methodological errors may have been introduced at several points in the digital workflow. Comparing scans in an engineering 3D software can introduce error from imperfect superimposition; the accuracy of the Geomagic software program has been validated previously by Toole et al. [34], who reported that the system quantified the degree of change accurately. Reliability was not formally established; repeating each measurement three times and averaging the results reduces random error but does not quantify it. Casts were produced by digital light-processing 3D printing in model resin, so printer accuracy, resin shrinkage, and post-curing dimensional change can also lead to errors. Digitizing the mounted casts with a desktop rather than an industrial scanner may introduce further error within the accepted accuracy range. Two features of the analysis are also exploratory rather than confirmatory. The equivalence margins were selected after the data had been inspected rather than being specified in advance, so the two one-sided tests indicate that an exploratory criterion was met and do not constitute a formal equivalence trial. The Bland–Altman limits of agreement pool repeated observations from the same participants and are not adjusted for within-participant correlation, so they describe the spread of the observed differences but should not be used inferentially. A prospectively registered margin and a repeated-measures agreement analysis are both required in the confirmatory study.

Finally, only the MIP was investigated. Capturing centric relation may be more demanding for the scanner algorithm and potentially more susceptible to gravity and chair inclination, and studies of centric relation registration with virtual records at different inclinations would be valuable. Against these limitations, the contribution of this work is to demonstrate a reproducible workflow, and it supplies the variance and agreement estimates needed to design an adequately powered equivalence trial. The present study adds a six-axis characterization across the full 90-to-180-degree range with a paired conventional comparator, and the protocol and effect-size estimates reported here can inform the design of a definitive trial.

### 4.2. Clinical Relevance

Within the limitations of this pilot investigation, virtual interocclusal records for capturing the mandibular position at MIP were insensitive to chair backrest inclination and met the exploratory post hoc equivalence criterion against conventional records in most rotational axes, which is consistent with their use as an alternative to conventional techniques. A small systematic vertical offset between the two workflows was nevertheless present, and its clinical relevance cannot be settled from these data. These results should be treated as preliminary and hypothesis-generating for populations similar to the healthy population studied rather than as a basis for changing practice.

## 5. Conclusions

In this pilot sample, dental chair backrest inclination between 90 and 180 degrees had no statistically detectable effect on the mandibular position recorded at MIP with either recording method. The conventional and virtual workflows differed by a small but systematic translational offset, most clearly along the Y axis, whereas most rotational comparisons met the exploratory post hoc equivalence criterion. These findings are preliminary and require confirmation in a larger, prospectively powered and randomized equivalence trial with a prospectively defined equivalence margin.

## Figures and Tables

**Table 1:** Per-axis linear mixed-effects models of the effect of recording method and chair inclination on mandibular position at MIP (primary analysis). Each row is a separate model fitted to one outcome, with participant as a random intercept.

Outcome	Analysis	Method Effect (95% CI)	*p*	p (Holm)	Inclination p (Holm)	Method × Inclination p (Holm)	Participant ICC
Translation X (mm)	Primary	+0.23 (+0.06, +0.40)	0.012	0.059	1.000	1.000	0.09
Translation Y (mm)	Primary	+0.27 (+0.19, +0.34)	<0.001	<0.001	1.000	1.000	0.42
Translation Z (mm)	Primary	−0.07 (−0.19, +0.06)	0.298	0.894	1.000	1.000	0.01
Rotation X (°)	Primary	+0.07 (−0.30, +0.45)	0.693	0.894	1.000	1.000	0.16
Rotation Y (°)	Primary	+0.34 (−0.13, +0.81)	0.159	0.635	1.000	0.981	0.03
Rotation Z (°)	Primary	+0.29 (−0.33, +0.90)	0.359	0.894	1.000	0.981	0.00
Translation X (mm)	Sensitivity	+0.31 (+0.22, +0.41)	<0.001	<0.001	1.000	1.000	0.37
Translation Y (mm)	Sensitivity	+0.25 (+0.18, +0.31)	<0.001	<0.001	1.000	1.000	0.56
Translation Z (mm)	Sensitivity	−0.07 (−0.20, +0.05)	0.263	0.917	1.000	1.000	0.01
Rotation X (°)	Sensitivity	+0.00 (−0.36, +0.37)	0.981	1.000	1.000	1.000	0.16
Rotation Y (°)	Sensitivity	+0.09 (−0.06, +0.24)	0.229	0.917	0.263	1.000	0.10
Rotation Z (°)	Sensitivity	−0.05 (−0.21, +0.12)	0.573	1.000	1.000	1.000	0.04

Each row is a separate linear mixed-effects model with participant as a random intercept, fitted to the 120- and 180-degree conditions in which both recording methods are observed. *p* values are from likelihood-ratio tests; p (Holm) is adjusted across the six outcomes. The sensitivity analysis excludes the two outlier records. CI, confidence interval; p (Holm), Holm–Bonferroni-adjusted *p* value across the six outcomes; ICC, proportion of residual variance attributable to between-participant differences [29].

**Table 2:** Descriptive statistics and linear mixed-effects estimates of 3D positional deviations in the translation and rotation axes for the conventional interocclusal record of MIP captured at 90-, 120-, and 180-degree dental chair backrest inclinations.

Chair Inclination	Translation (mm)						Rotation (°)					
	X		Y		Z		X		Y		Z	
	Mean	SD	Mean	SD	Mean	SD	Mean	SD	Mean	SD	Mean	SD
CR90	0.47	0.32	0.56	0.72	0.64	1.75	−0.35	0.40	0.01	0.23	0.02	0.43
CR120	0.33	0.20	0.25	0.15	0.03	0.31	−0.12	0.53	−0.04	0.33	−0.06	0.20
CR180	0.30	0.29	0.27	0.21	−0.05	0.23	0.13	1.12	0.17	0.30	0.01	0.35
180° vs. 90° (95% CI)	−0.18 (−0.32, −0.03)		−0.29 (−0.61, +0.03)		−0.69 (−1.55, +0.17)		+0.48 (+0.02, +0.95)		+0.16 (+0.00, +0.32)		−0.00 (−0.28, +0.27)	
p (Holm)	0.317		0.536		0.536		0.536		0.245		0.829	

SD, standard deviation; CR90, CR120, CR180: conventional interocclusal records obtained at 90, 120, and 180 degrees, respectively. Inferential values are from a linear mixed-effects model with participant as a random intercept, is adjusted across the six outcomes.

**Table 3:** Descriptive statistics and within-participant contrasts of 3D positional deviations in the translation and rotation axes for the virtual interocclusal record of MIP captured at 90-, 120-, and 180-degree dental chair backrest inclinations.

Chair Inclination	Translation (mm)						Rotation (°)					
	X		Y		Z		X		Y		Z	
	Mean	SD	Mean	SD	Mean	SD	Mean	SD	Mean	SD	Mean	SD
VR90	0.00	0.00	0.00	0.00	0.00	0.00	0.00	0.00	0.00	0.00	0.00	0.00
VR120	0.02	0.09	0.00	0.11	0.05	0.08	0.02	0.24	−0.08	0.15	0.07	0.22
VR180	0.16	0.47	0.00	0.16	0.06	0.09	−0.15	0.45	−0.49	1.55	−0.71	2.05
180° vs. 120° (95% CI)	+0.13 (−0.25, +0.51)		−0.00 (−0.15, +0.14)		+0.03 (−0.04, +0.11)		−0.17 (−0.61, +0.27)		−0.43 (−1.63, +0.76)		−0.77 (−2.39, +0.85)	
p (Holm)	1.000		1.000		1.000		1.000		1.000		1.000	

SD, standard deviation; VR90, VR120, VR180: virtual interocclusal records obtained at 90, 120, and 180 degrees, respectively.

**Table 4:** Descriptive statistics of 3D positional deviations for the conventional and virtual interocclusal records at each chair inclination, with the Holm-adjusted method effect on each axis. Two one-sided test (TOST) equivalence results are reported separately in Table 5.

Chair Inclination	Translation (mm)						Rotation (°)					
	X		Y		Z		X		Y		Z	
	Mean	SD	Mean	SD	Mean	SD	Mean	SD	Mean	SD	Mean	SD
CR90	0.47	0.32	0.56	0.72	0.64	1.75	−0.35	0.40	0.01	0.23	0.02	0.43
CR120	0.33	0.20	0.25	0.15	0.03	0.31	−0.12	0.53	−0.04	0.33	−0.06	0.20
CR180	0.30	0.29	0.27	0.21	−0.05	0.23	0.13	1.12	0.17	0.30	0.01	0.35
VR90	0.00	0.00	0.00	0.00	0.00	0.00	0.00	0.00	0.00	0.00	0.00	0.00
VR120	0.02	0.09	0.00	0.11	0.05	0.08	0.02	0.24	−0.08	0.15	0.07	0.22
VR180	0.16	0.47	0.00	0.16	0.06	0.09	−0.15	0.45	−0.49	1.55	−0.71	2.05
Method effect p (Holm)	0.059		<0.001		0.894		0.894		0.635		0.894	

**Table 5:** Within-participant difference between conventional and virtual interocclusal records at each chair inclination, with two one-sided test (TOST) results against the post hoc equivalence margins of ±0.5 mm and ±1.0 degrees (primary analysis). “Yes” indicates that the 90% confidence interval lays entirely within the post hoc margin, that is, that the exploratory equivalence criterion was met.

Outcome	Inclination	*n*	C–V (95% CI)	dz	p (Holm)	90% CI	Equivalent
Translation X (mm)	90°	10	+0.47 (+0.24, +0.70)	+1.47	0.019	(+0.29, +0.66)	No
Translation X (mm)	120°	10	+0.31 (+0.18, +0.45)	+1.67	0.008	(+0.20, +0.42)	Yes
Translation X (mm)	180°	9	+0.18 (−0.25, +0.61)	+0.32	1.000	(−0.17, +0.53)	No
Translation Y (mm)	90°	10	+0.56 (+0.05, +1.07)	+0.78	0.469	(+0.14, +0.98)	No
Translation Y (mm)	120°	10	+0.25 (+0.16, +0.34)	+2.00	0.003	(+0.18, +0.33)	Yes
Translation Y (mm)	180°	9	+0.30 (+0.11, +0.49)	+1.20	0.103	(+0.14, +0.45)	Yes
Translation Z (mm)	90°	10	+0.64 (−0.61, +1.89)	+0.37	1.000	(−0.38, +1.65)	No
Translation Z (mm)	120°	10	−0.02 (−0.26, +0.22)	−0.05	1.000	(−0.21, +0.17)	Yes
Translation Z (mm)	180°	9	−0.12 (−0.35, +0.11)	−0.41	1.000	(−0.31, +0.06)	Yes
Rotation X (°)	90°	10	−0.35 (−0.64, −0.07)	−0.88	0.297	(−0.58, −0.12)	Yes
Rotation X (°)	120°	10	−0.13 (−0.52, +0.26)	−0.24	1.000	(−0.45, +0.18)	Yes
Rotation X (°)	180°	9	+0.34 (−0.60, +1.28)	+0.28	1.000	(−0.42, +1.10)	No
Rotation Y (°)	90°	10	+0.01 (−0.16, +0.18)	+0.04	1.000	(−0.13, +0.15)	Yes
Rotation Y (°)	120°	10	+0.04 (−0.15, +0.22)	+0.14	1.000	(−0.11, +0.19)	Yes
Rotation Y (°)	180°	9	+0.67 (−0.55, +1.89)	+0.42	1.000	(−0.31, +1.65)	No
Rotation Z (°)	90°	10	+0.02 (−0.29, +0.32)	+0.04	1.000	(−0.23, +0.27)	Yes
Rotation Z (°)	120°	10	−0.13 (−0.31, +0.04)	−0.54	1.000	(−0.27, +0.01)	Yes
Rotation Z (°)	180°	9	+0.75 (−0.91, +2.41)	+0.35	1.000	(−0.59, +2.09)	No

**Table 6:** Bland–Altman agreement between conventional and virtual interocclusal records, pooling the 120- and 180-degree conditions in which both recording methods are observed. The pooled differences are not independent, because the 19 (primary) and 18 (sensitivity) differences arise from 10 participants; the limits of agreement are unadjusted for within-participant correlation and are descriptive only.

Outcome	Analysis	*n*	Bias, C–V (95% CI)	SD of Differences	Lower LoA (95% CI)	Upper LoA (95% CI)
Translation X (mm)	Primary	19	+0.25 (+0.06, +0.44)	0.40	−0.53 (−0.87, −0.20)	+1.03 (+0.70, +1.37)
Translation Y (mm)	Primary	19	+0.27 (+0.18, +0.36)	0.19	−0.10 (−0.25, +0.06)	+0.64 (+0.49, +0.80)
Translation Z (mm)	Primary	19	−0.07 (−0.22, +0.08)	0.31	−0.68 (−0.94, −0.42)	+0.54 (+0.28, +0.80)
Rotation X (°)	Primary	19	+0.09 (−0.36, +0.54)	0.94	−1.74 (−2.52, −0.96)	+1.93 (+1.14, +2.71)
Rotation Y (°)	Primary	19	+0.34 (−0.20, +0.88)	1.12	−1.86 (−2.80, −0.92)	+2.53 (+1.60, +3.47)
Rotation Z (°)	Primary	19	+0.29 (−0.45, +1.02)	1.52	−2.69 (−3.96, −1.42)	+3.26 (+1.99, +4.53)
Translation X (mm)	Sensitivity	18	+0.32 (+0.21, +0.44)	0.24	−0.14 (−0.34, +0.07)	+0.79 (+0.58, +0.99)
Translation Y (mm)	Sensitivity	18	+0.25 (+0.17, +0.32)	0.15	−0.05 (−0.18, +0.08)	+0.54 (+0.41, +0.68)
Translation Z (mm)	Sensitivity	18	−0.07 (−0.23, +0.08)	0.32	−0.70 (−0.97, −0.42)	+0.55 (+0.28, +0.83)
Rotation X (°)	Sensitivity	18	+0.03 (−0.43, +0.49)	0.92	−1.77 (−2.56, −0.98)	+1.83 (+1.04, +2.62)
Rotation Y (°)	Sensitivity	18	+0.09 (−0.08, +0.27)	0.35	−0.59 (−0.89, −0.29)	+0.78 (+0.47, +1.08)
Rotation Z (°)	Sensitivity	18	−0.05 (−0.25, +0.15)	0.40	−0.83 (−1.18, −0.49)	+0.73 (+0.39, +1.07)

C–V, conventional minus virtual; LoA, 95% limits of agreement (bias ± 1.96 SD). Confidence intervals for the limits are estimated as bias ± 1.96 SD ± t(0.975, n−1) × √(3 SD^2^ / n). dz, standardized paired effect size; p (Holm), Holm–Bonferroni-adjusted across the 18 comparisons.

## Data Availability

The data that support the findings of this study are available from the corresponding author upon reasonable request.
